# Complications of Intrathecal Baclofen Pump Therapy: An Institutional Experience from Saudi Arabia

**DOI:** 10.3390/healthcare11212820

**Published:** 2023-10-25

**Authors:** Ahmad Zaheer Qureshi, Hasan Shacfe, Amara Ilyas, Saeed Bin Ayaz, Khalid Yousef Aljamaan, Imad Saeed Moukais, Mohammed Jameel, Waqas Sami, Sami Ullah

**Affiliations:** 1 Department of Physical Medicine and Rehabilitation, King Fahad Medical City, Riyadh 11525, Saudi Arabia; 2 Department of Physical Medicine and Rehabilitation, King Fahad Specialist Hospital, Damam 32553, Saudi Arabia; 3 Department of Rehabilitation Medicine, Sheikh Khalifa Bin Zayed Al Nahyan Hospital, Muzaffarabad 13100, Pakistan; 4 Medical Rehabilitation Department, King Fahad Hospital, Hofuf 36364, Saudi Arabia; 5 Department of Pain Medicine, King Fahad Medical City, Riyadh 11525, Saudi Arabia; 6 Department of Pre-Clinical Affairs, College of Nursing, QU Health, Qatar University, Doha P.O. Box 2713, Qatar; waqas@qu.edu.qa; 7 Department of Physical Medicine and Rehabilitation, Qatar Rehabilitation Institute, Doha P.O. Box 3050, Qatar

**Keywords:** intrathecal baclofen, spasticity, complications, outcomes, Saudi Arabia

## Abstract

The intrathecal baclofen pump (ITB) is one of the advanced treatment options in the management of spasticity. This retrospective cohort study was conducted to identify the complications of ITB treatment at a tertiary care rehabilitation facility. Various demographic and technical factors were analyzed, which are less often reported in the literature. All patients with ITB who had their refill at the ITB clinic between November 2019 and March 2020 were included. Of 48 patients, 17 patients had 18 (37.5%) ITB-related complications. Catheter-related complications were most common, whereas loss of efficacy (16.7%) and baclofen withdrawal (14.5%) were the most common outcomes of complications. Only catheter occlusion had a significant relationship with the pattern of spastic quadriparesis (*p* = 0.001). Gender, rehabilitation diagnosis, patients’ residence, and facility of ITB placement did not have significant association. Similarly, age, distance from hospital, disease onset, ITB therapy duration, and baclofen dose were not statistically significant in relation to ITB-related complications.

## 1. Introduction

The intrathecal baclofen pump (ITB) is one of the advanced treatment options in the management of spasticity; however, there are various complications associated with it. ITB complications are generally related to operative or technical procedures and device–catheter system malfunction [1,2,3,4,5,6]. Technical complications may be related to the pump, catheter, surgery, or refill techniques. Pump-related surgeries may be complicated with bleeding, infections, seroma, or cerebrospinal fluid leakage, whereas catheter-related problems can be caused by blockage, kinking, fracture, disconnect, tear, migration, or dislodgement [2,3]. Catheter-related complications are reported to be the highest, whereas pump malfunction is less commonly reported [4]. In addition to device malfunction, human error (such as programming or refill error) can also lead to adverse outcomes [5,6]. Subsequent pharmacological complications, like loss of efficacy, withdrawal, or overdose of baclofen, may impose serious effects on patients [7,8]. Even though the outcomes of ITB complications are reported to be associated with morbidity and mortality, there have been growing trends in the use of ITB across various health systems [9]. ITB management involves a skilled and specialized multidisciplinary team [4]. The requirements of running a safe and effective program are not only limited to clinical expertise. Patient selection, payer system, access to specialized care, support structure, and socioeconomic factors are important considerations in ITB service [10]. Since the clinical expertise, health system, patient population, and institutional practices vary around the globe, it is important to study local practices and outcomes associated with ITB management.

There is a paucity of local data regarding ITB practices [8,11]. Saudi Arabia has a population of approximately 34 million, which is distributed across 13 provinces [12]. Currently, only four hospitals are providing intrathecal baclofen service, out of which three are in the capital city of Riyadh, whereas one is in the Eastern Region. Many patients with ITB are residents of regions with limited or no access to specialized ITB care. Also, it is not uncommon for patients to be placed on ITB in foreign countries without preemptive plans of continued treatment upon their return to Saudi Arabia. Similarly, patient selection and pump placement may be carried out at a facility different than the one handling continued care. Hence, it remains important to analyze the complications associated with ITB management, which can help to identify gaps and facilitate the adaptation of pertinent strategies to improve service delivery.

This study was conducted to identify the complications of ITB treatment at a tertiary care rehabilitation facility and highlight the pertinent challenges involved in ITB care.

## 2. Materials and Methods

### 2.1. Type

This study is a retrospective cohort study.

### 2.2. Setting

The setting is the intrathecal baclofen pump clinic, Rehabilitation Hospital of King Fahad Medical City (KFMC), Riyadh. The Rehabilitation Hospital at KFMC is the largest ministry of health tertiary care rehabilitation facility in the country, receiving referrals from all over the Kingdom and offering comprehensive rehabilitation services. The ITB service involves a multidisciplinary team, including rehabilitation physicians, neurosurgeons, rehabilitation nursing, and therapists as core team members. The ITB clinic at KFMC is a weekly clinic where patients follow-up for review of spasticity and ITB pump management.

### 2.3. Inclusion Criteria

All patients with ITB who had their pump refill at the ITB clinic between November 2019 and March 2020 were included in this study.

### 2.4. Exclusion Criteria

Patients who died during hospital admission or were transferred back to the acute care were excluded.

### 2.5. Procedure

This study was approved by the institutional review board of KFMC, Riyadh. A chart review was carried out for all patients who fulfilled the inclusion criteria. Information was obtained regarding diagnoses, demographics, baclofen dose, device details, and pattern of spasticity. The data regarding complications included pump site infection, programming error, procedural complications, and catheter malfunction. The outcomes were recorded as withdrawal, overdose, or loss of efficacy. Information was collected regarding any pump or catheter-related surgery as a result of complications. Additionally, appointment compliance and follow-up with the primary rehabilitation doctor were reviewed at the last refill clinic visit.

### 2.6. Statistical Analysis

The Statistical Package for Social Sciences (SPSS) version 22 was used for the statistical analysis. A descriptive analysis was carried out by calculating numbers and percentages for the categorical data. The scale data were analyzed for normality using the Shapiro–Wilk test. Means and standard distributions were calculated for the normally distributed scale data, whereas medians and interquartile ranges (IQR) were recorded for the scale data that were not normally distributed. The chi-square/Fischer’s Exact tests were used to analyze the relationship between different complications and gender, rehabilitation diagnoses, spasticity pattern, living area of the patients, and place of placement of the ITB. A logistic regression was performed to ascertain the effects of age, distance from Riyadh city (km), time since onset of primary disease (months), duration of ITB placement (months), and baclofen dose (µg/day).

## 3. Results

**Figure 1:**
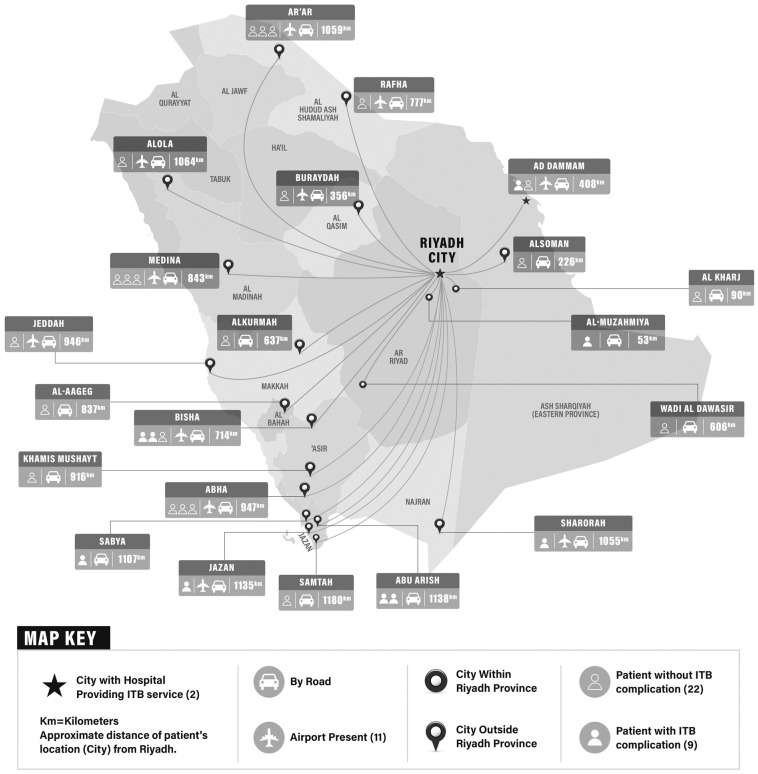
Geographic distribution of the patients with an intrathecal baclofen pump outside Riyadh City.

A total of 48 patients met the criteria for this study. The median age of the patients was 30 years (range: 16–69 years). Out of 48 patients, 36 (75%) were male, and 12 (35%) were female (Table 1). The most common diagnosis was spinal cord injury (SCI) (66.7%, n = 32). All patients were on ITB therapy for the management of spasticity except for one patient with cerebral palsy who had dystonia. A total of 64.6% (n = 31) of the patients with ITB were from outside Riyadh city with most residing at locations 400 km to 1200 km from Riyadh city (Figure 1). The spinal catheter tip was at the thoracic vertebrae level in 23 (47.92%) patients, while in 20 (41.7%) patients, the level of the spinal catheter tip was not known. The other characteristics related to the patients and ITB in our cohort are outlined in Table 1.

The median daily dose of baclofen at the initial ITB pump placement was 300.2 µg/day with minimum and maximum dosing values of 48.96 µg/day and 661.30 µg/day, respectively; however, at the last clinic visit, there was an increase in the median daily dose to 325.45 µg/day (maximum: 661.30 µg/day; minimum: 48.96 µg/day) (Table 2). The mean time since last refill was approximately 4 months, which approximately remained the same for future refills. The mean time since disease onset was approximately 15 years. The mean time for patients while on ITB therapy was approximately 10 years. The median duration (IQR) of the last appointment with the primary rehabilitation physician (other than the pump manager) was approximately 23 (29) months. Eleven patients did not have a primary rehabilitation physician, and they only followed up with the rehabilitation physician managing the pump.

Seventeen patients had 18 ITB-related complications, out of which one patient had complications twice. The frequency of complications was 37.5% (Table 3). Eleven patients with ITB-related complications had been on ITB therapy for more than seven years. The most common complications were catheter-related, which constituted one-third (33.3%) of all the complications, out of which four were catheter occlusions and two were catheter breakages. Programming error occurred in four out of eighteen patients (22.2%). Of all the patients who had ITB-related complications, 11 (64.7%) had their pumps placed at other institutes. Seven out of seventeen patients had ITB withdrawal, out of which four were from outside Riyadh province.

Overall, the most common adverse outcome in our study population was loss of efficacy (16.7%), which constituted 44.4% of all ITB-related complications. Three patients with loss of efficacy had catheter-related problems for which they underwent catheter replacement. Additionally, two patients underwent ITB exploratory surgery to investigate the loss of efficacy, while the remaining three only required fluoroscopic evaluations, which were non-conclusive. The second most common adverse outcome was baclofen withdrawal (14.5%), which constituted 38.8% of all ITB-related complications. The majority of patients with ITB withdrawal had a diagnosis of SCI (71.4%). Catheter occlusion and programming error were found to be the most common reasons for withdrawal for which catheter replacements were carried out. Three patients had withdrawal due to programming error, while one withdrawal occurred due to a pump malfunction that required pump replacement. Two patients had pump site infections, out of which one was associated with withdrawal, while the other one required pump replacement. One patient had a cerebrospinal fluid culture positive for staphylococcus infection, requiring treatment with cloxacillin. Out of the two cases who had a baclofen overdose, one was due to a programming error, while the other one occurred after fluoroscopic evaluation with contrast through the catheter access port (CAP), requiring intensive care. A total of 10 surgical procedures were carried out, indicating that approximately one in five patients required surgical intervention in our study population. Approximately half (55.5%) of the ITB-related complications required surgical intervention.

Following the chi-square/Fischer’s Exact Test analysis, only catheter occlusion showed a significant relationship with spastic quadriparesis (*p* = 0.001) (Table 4). The rest of the test variables, i.e., gender, rehabilitation diagnoses, spasticity pattern, living area of the patients, and place of placement of ITB pump, did not show any significant correlation with the different ITB-related complications. Moreover, the logistic regression model did not identify age, distance from Riyadh city, time since onset of primary disease, duration of ITB placement, or baclofen dose as possible risk factors for the development of complications (Table 5).

## 4. Discussion

### 4.1. Patient Characteristics

(a) Pattern of spasticity and functional impairments

All patients in our study population had pump placements for spasticity except one patient with cerebral palsy who had dystonia. Though other institutional studies report the use of ITB in dystonia, ITB is less frequently used in dystonia as compared to spasticity. There may be a lack of studies demonstrating improvements in dystonia with the use of ITB, the need for higher doses, and the risks of a dystonic storm with sudden discontinuation [1,13,14]. On the other hand, the ITB-related complications in patients with dystonia are similar to what is reported in patients with spasticity [15]. It is interesting to note that the patients with ITB in our study had complications that may be expected to be less frequent in patients with controlled spasticity. For example, 87.5% of the patients had bladder and bowel incontinence, while approximately one-third of the patients had pressure ulcers (35.4%) and contractures (33.3%). Though this could be multifactorial, it may be attributed to SCI as the most common diagnosis (66.7%) or because the majority of patients had spastic quadriparesis. This emphasizes the need for multidisciplinary rehabilitation services beyond pump management. It is important for services running ITB programs to ensure that relevant specialties are on board when problems beyond spasticity arise; for example, plastic surgery, orthopedics, and urology are integral services for individuals with long-term disabilities.

(b) Access to continued care

Out of the patients from outside Riyadh, three patients were from the Eastern Region, which has a hospital running its ITB service, but the patient preferred to follow-up at our institute. Since half of the patients who had ITB-related complications were also from outside Riyadh, the potential morbidity or mortality associated with ITB complications brings attention to the need for access to emergency care locally. Currently, only four hospitals are providing ITB services across the country. They include King Fahad Medical City (Riyadh), Prince Sultan Military Medical City (Riyadh), Prince Sultan Bin Abdulaziz Humanitarian City (Riyadh), and King Fahad Specialist Hospital Dammam (Eastern Region). All these hospitals offer inpatient and outpatient comprehensive rehabilitation services and physical medicine, and rehabilitation physicians are involved in the care of patients with ITB. There is no national registry of patients with ITB at present, and patients are observed to shift their care from one hospital to another. Half of the patients with ITB-related complications in our study had their pumps placed at another facility. The reasons for the transfer of care were not analyzed in our study, but to our observation, the usual causes include lack of funding to continue ITB service, closure of ITB service, or ITB placement at a facility outside Saudi Arabia.

(c) Challenges of transfer of care to new institute and its implications

After pump placement at one facility, some patients prefer to establish ITB care with their primary rehabilitation physician at another institute who had been involved in their holistic rehabilitation care previously. Challenges ensue when the primary rehabilitation team may not be aware of the pump placement until notified about it only when the pump has already been placed at another institute. In such situations, there could be insufficient information on the treatment tried for spasticity before pump placement. Similarly, continued treatment at a new facility could be challenging due to lack of clarity on the process of patient selection and appropriateness of ITB therapy for a particular patient. Additionally, there could be deficient information regarding the brand name of baclofen and details of ITB trials. Pump information and most catheter details may be retrievable if it is a Medtronic drug infusion pump; however, the information on catheter tip level may be missing as this information is not auto-retrievable through a programmer. This was a major concern that we identified in our study, since catheter tip level could not be retrieved from chart review in a considerable number (41.7%) of patients. This is primarily because many of the patients who had their pump placements at other institutes established care at our center for routine refills, not requiring imaging for spinal catheters unless there were concerns of complications. Documentation from previous hospitals did not include this information; however, this is an area of improvement. When not known, spinal imaging needs to be included as a part of the initial workup to determine the catheter tip level when a patient with ITB establishes care at a new facility. Knowledge of the catheter tip level is important to know the effectiveness of spasticity treatment. A cervically positioned catheter tip could relieve spasticity in the upper limbs due to a higher cervical concentration of baclofen. At the same time, a bolus or high baclofen doses can affect respiratory function if the catheter tip is high, which could be concerning in patients with impaired ventilation, such as individuals with cervical SCI. Imaging of the catheter can also help to plan safe spinal taps or epidural anesthesia when needed.

Due to the above-mentioned challenges, a pump manager new to the patient who had his or her pump placement elsewhere may not only have to face a cumulative challenge related to patient safety but may also have potential medicolegal implications. This can lead to a clinical dilemma when a rehabilitation physician who had neither been involved in decision-making nor had considered a referral for pump placement may eventually be expected to undertake ITB care post-pump placement carried out at another facility by another provider. So far, we have neither come across a patient with a drug infusion pump other than Medtronic nor is there another brand label currently operating in the country; however, a problematic situation can arise if a patient abroad is placed on an intrathecal drug infusion pump other than Medtronic and returns to Saudi Arabia.

### 4.2. Importance of Patient Selection in ITB Therapy

It is of paramount importance to determine appropriate patient selection based on the reliability and capability of the patient and their families to be able to ensure compliance with instructions critical for patient safety [10]. Psychological attributes, socio-economic factors, access to care, support services, and availability of expertise remain important factors in patient selection [16,17]. The treating teams should not only make management decisions on the clinical attributes, but rather non-clinical factors should always be prioritized, given their potential to affect the continuity of safe and effective ITB treatment. For patients opting for ITB management, the statistics regarding expected adverse outcomes published in the literature can help to improve informed decision-making.

### 4.3. Complications

The frequency of complications in our study was similar to what has been reported in Japan (37%) [7]. The literature review shows that 20–50% of patients with ITB can have ITB-related complications [18,19,20,21,22].

### 4.4. Complications—Catheter Related

Catheter-related complications remain the most common cause of ITB-related problems, which can occur in up to 40% of patients with ITB [18,21,23]. Catheter malfunction constituted 33% of the ITB-related complications in our study, which is similar to what was published (37%) in a meta-analysis of 2264 patients [18]. This raises a high suspicion of catheter-related problems while investigating ITB-related concerns but does not necessitate the need for a catheter workup as the first step during ITB troubleshooting. Various protocols have been recommended to investigate ITB-related problems; however, the approach needs to be individualized for each case [21,23,24,25]. At the same time, the failure to identify a catheter-related problem does not exclude a catheter malfunction. Plain imaging and fluoroscopic myelography may be non-conclusive, and further evaluations may be required, such as computerized tomography (CT) myelogram, non-contrast CT, magnetic resonance imaging (MRI), lumbar puncture, and isotopic scintigraphy [21,23,24]. The use of catheters other than Ascenda^®^ catheters has been associated with fewer catheter-related complications [22].

### 4.5. Complications—Infection

The incidence of infection related to ITB is reported to be 3.2–27.5% [26]. In our study, two patients had pump site infections, out of which one was associated with withdrawal, while the other one was treated for local infection only. Similarly, two post-surgical pump site infections were reported in 11 years of the observational period in a hospital-based study in Austria [22]. A wide incidence range of infection in the literature may not only be due to procedural or institutional factors but also pump exteriorization may occur long after pump placement due to trauma or pressure injury over the pump site, leading to infection [27]. Hence, factors unrelated to pump, procedures, or spasticity play a crucial role in the successful long-term continuation of ITB therapy.

Approximately one-fifth of the patients with ITB in our study underwent surgical interventions, whereas approximately half of the ITB-related complications required surgical intervention. In a study on 195 patients with ITB, the surgical procedures for complications included 7 pump revisions, 48 catheter revisions, and 41 wound revisions [26]. Since ITB therapy is intended to be a long-term treatment in most cases, the chances of complications persist throughout the treatment period. In our study, 11 out of 17 patients with ITB-related complications had been on ITB therapy for more than 7 years. In a 7-year retrospective study on 243 patients on intrathecal baclofen and opioid therapy, CSF leakage and deep infections were reported in 19% and 5% of patients, respectively, which led to an increase in readmissions and hospital stays [9].

In summary, the infections related to ITB therapy can be attributed to the immediate post-surgical period or delayed infections due to pump exteriorization, pressure injury, or trauma. Peri-operative care has to be optimized for post-operative surgical infections. For patients admitted for pump- or catheter-related surgery, routine screening can be introduced for MRSA *(methicillin-resistant Staphylococcus aureus)* and ESBL *(Extended spectrum beta-lactameses)* producing bacteria. Standard infection control protocols and infectious disease consultations can help to ensure peri-operative care. For delayed infections related to trauma or pressure injury over the pump site, patients and families have to be counselled extensively for pump care at home and should be able to seek immediate care to ensure early intervention. A minor blister, swelling, or bruise over the pump or connector sites should not be ignored and should be brought to the knowledge of the treating physician.

### 4.6. Outcome—Loss of Efficacy

In our study, the most common outcome of ITB-related complication was the loss of baclofen efficacy for which catheter malfunction was identified in three patients. The exact cause of the loss of efficacy could not be determined in the rest of the five patients, out of which three patients underwent fluoroscopic myelography, while two had surgical exploration when fluoroscopic myelography was non-conclusive. Other imaging evaluations were not carried out on these patients. Surgical exploration has its limitations, as it cannot directly visualize the intrathecal component of the catheter for which imaging may still be required. Alternatively, the catheter needs to be replaced in the case of a high suspicion of catheter malfunction. The second most common complication was baclofen withdrawal in our study. Catheter occlusion was found to be the most common reason for withdrawal, for which catheter replacements were carried out. This brings attention to the need for expertise in the radiological evaluation of the pump and catheter systems. Radiologists may not be well-versed in the pump technicalities and operational aspects of the device systems. The dye testing protocol via CAP could potentially lead to a baclofen overdose, as in one of our cases. Baclofen overdose is a rare complication that requires urgent management and intensive care. The detection of the earliest symptoms and signs of baclofen overdose or withdrawal may be challenging in a radiology setting, where staff may not be familiar with emergencies related to ITB. Similarly, clinical expertise across all medical, surgical, nursing, and allied health professionals needs to be ensured at the time of introducing intrathecal drug delivery programs across an institution or health system [16,17]. Feller et al. observed catheter-related complications in 7.1% of patients. They used reinforced catheters, which led to low catheter-related complications as compared to the previous studies [2]. This could be a potential alternate in case catheter replacement is not possible.

The differential diagnosis of withdrawal could be challenging in patients with SCI, as it may mimic autonomic dysreflexia, meningitis, hyperthermia, neuroleptic malignant syndrome, serotonin reuptake syndrome, and sepsis [28,29]. A total of 40% of the patients with ITB complications were reported to be associated with withdrawal and overdose [29]. Watve et al. reviewed 23 articles on ITB withdrawal and reported that 40% of the withdrawal complications were due to catheter-related issues [30]. On the other hand, ITB treatment is commonly used in patients with SCI [5,24,29,31]. Hence, teams managing ITB need to be well-versed with ITB withdrawal as well as the autonomic aspects of SCI. Emergency physicians at institutes managing ITB must be familiar with ITB complications, especially withdrawal. It is imperative that the ITB managing teams are available 24/7, and patients have access to the nearest facility offering specialized ITB care. Of the seventeen patients who had ITB-related complications, nine (53%) had their pumps placed at other institutes. Seven out of eighteen patients had ITB withdrawal, out of which four were from outside Riyadh city. Given that more than half of the patients who had withdrawal were from outside Riyadh city (400–1200 km away), early identification of complications and coordination of their care at a local hospital or the primary pump managing facility remain crucial. This requires a 24/7 helpline or access to a program coordinator, which could be challenging after working hours or on weekends; however, education regarding precautions and withdrawal symptoms needs to be carried out on a regular basis with patients and families. A case study reported that a 31-year-old Saudi male with SCI had baclofen withdrawal twice: once due to missing an appointment, and the second due to the non-availability of his primary pump manager; so, the patient and family decided to wait and did not seek care with another physician or institute as per the case report [8]. This is a classic local example of an ITB-related complication, which merely reflects the tip of an iceberg. The lack of reporting of such cases can result in an inability to determine the actual severity of the problem and its impact. A delay in baclofen refill is a simpler yet preventable problem, the outcomes of which could be disastrous. It can be prevented by proper patient selection, patient and caregiver education, scheduled pump refilling, and timely access to specialized expertise [8].

### 4.7. Factors Associated with ITB Complications and Outcomes

Regarding the relationship of different complications with the variables of this study, only catheter occlusion established a significant association with the spasticity quadriparesis. The remaining variables of gender, rehabilitation diagnoses, location of the patients’ home, and place of placement of the ITB pump did not have a significant association. Similarly, age, distance from Riyadh (km), time since onset of primary disease (months), duration of ITB placement (months), and baclofen dose (µg/day) did not prove to be risk factors for the development of any type of ITB-related complication. Other investigations in the literature have also observed similar results. Gender, age, level of ambulation, gastrostomy tube, and pump years were found to have no significant influence on the development of a complication in children by Bonouvrié et al. [3]. Only the type of catheter was identified as a significant risk factor with an odds ratio of 3.75 (95%CI: 1.30–10.83). Another pediatric cohort identified young age, wound dehiscence, and the number of revisions as independent risk factors for infection related to ITB [32]. Potential risk factors described in other studies were the presence of dystonia and age [26,33]. It should be highlighted that the studies on complications in ITB are frequently challenging to compare because of the use of different terminologies to describe the complications and variability of methodology. For example, withdrawal is generally an outcome of an underlying cause, like a catheter break; however, both are described as complications in various studies. To avoid this, we gathered the data discretely to show the cause-and-effect relation for each ITB-related complication (Table 3).

### 4.8. ITB Therapy and Cost Impact—Regional Considerations

Though the clinical effectiveness of ITB treatment has been well established, there are mixed results when it comes to the cost-effectiveness. A study on complications of ITB in children reported an increased hospital length of stay of up to 4 days and an increase in the mean cost from $29,431 to $37,081 [34]. It has also been observed that ITB treatment increases the financial cost as compared to that of other interventions [35,36]. The cost-per-patient up to 1 year of treatment was found to be three times more for patients on ITB treatment as compared to those who underwent selective posterior rhizotomy [37]. ITB treatment was found to be cost-effective as compared to conservative and other surgical interventions in the French population, which reported an ITB complication rate of only 10% [36]. It is important to note that these cost analysis studies were reported from western health systems; however, no such studies have been conducted in health systems in countries like Saudi Arabia, United Arab Emirates, or Qatar, where payer systems are different, and public health is heavily funded by the government. In Saudi Arabia, the additional cost is attributed to support services for a routine clinic visit to alleviate the financial burden of patients visiting the hospital from distant locations. In addition to medical care, various services are offered by the government, which may include travel expenses for appointments. A routine ITB refill may also include the need to address medical concerns that could be otherwise managed at a primary care facility; however, patients generally tend to rely on tertiary care hospitals and may wait for the “all-in-one” visit. The impact of the lack of local specialized ITB care services and subsequent reliance on distant tertiary care specialized hospitals needs to be evaluated to determine the actual cost-effectiveness of ITB therapy in regional health systems. Beyond cost, the burden of travel is an important consideration, especially for patients who are dependent and require full-time assistance of a caregiver. The burden of travel was reported in five out of ten patients in a study from the United States with fatigue as the main contributing factor [35]. Home-based ITB care is an alternate strategy to address these concerns [10,38].

### 4.9. Future Directions and Recommendations

Given that there is a lack of specialized rehabilitation services in the country, hospitals running ITB programs should be able to provide 24/7 ITB services with an on-call ITB team coverage. Improving the expertise at local facilities is of utmost importance, especially for patients from remote areas who have difficult access to urgent care. Measures to ensure the prevention of post-operative infections need to be ascertained. Continual counselling, education, and close follow-up with patients in the community remain a hallmark to prevent or detect problems related to ITB. There could be potential medico-legal complexities in case complications of ITB that are a sequel of recent ITB management or an encounter at another institute. Hence, a patient who undergoes ITB placement at a particular center needs to be facilitated to continue care at the same institute. The criteria for ITB therapy need to be standardized, and a consensual agreement in this regard can be achieved by involving stakeholders from different clinical disciplines at a national level. Payers, device manufacturers, suppliers, and emergency response teams need to be taken onboard in policy making. Specific competencies for clinicians involved in ITB care is important. Currently, there is no standardized process at the level of the Ministry of Health to determine institutional readiness to provide holistic ITB services. Stringent institutional and national policies need to be undertaken to address this challenge. Such initiatives have been undertaken by various national bodies and organizations globally and can be replicated locally after improvisation [10,17,38,39,40]. The introduction of national policies prior to the approval of intrathecal device programs in healthcare facilities can set standardization and promote safe practices. We recommend creating a panel of experts at the level of the Ministry of Health to review the readiness of a facility interested in establishing ITB services and also to provide the necessary support to facilitate service provision across various regions of the country.

Furthermore, the nomenclature of “complications” and “outcomes” related to ITB therapy has to be standardized. The terms have been interchangeably used in the published literature. This is of utmost importance to generate reliable data and will help in ensuring standardized practice measures.

### 4.10. Limitations

The retrospective assessment of the data has to be taken into account. Several patients had their ITB pump placed at another facility before establishing care with our institute, limiting access to the data at the other facilities. Patients who had established care at another facility were excluded from this study due to the difficulty in accessing records and documentation. One of the other limitations of this study is the small sample size, which prevents inferential statistics. A multicenter study at the national level can help to determine the extent of problems associated with ITB therapy in the region and facilitate development of strategies and policies.

## 5. Conclusions

A high suspicion for catheter-related problems needs to be considered while investigating ITB-related problems, as catheter malfunction was the most common complication in our study. This could be of particular importance in patients with spastic quadriparesis. To investigate the loss of efficacy, advanced radiological techniques need to be incorporated in addition to fluoroscopy. Though pump placements at other facilities and the distance of the patient from the hospital providing ITB services are obvious challenges, especially in the case of emergency situations, our study did not demonstrate any significant statistical correlation with ITB-related complications. National strategies need to be adapted to establish uniform policies across various institutes offering ITB services.

## Figures and Tables

**Table 1:** Categorical data related to the patients and intrathecal baclofen pump.

Variables	n (%)	Variables	n (%)
**Gender**		**Pump placement**	
Male	36 (75)	King Fahad Medical City	23 (47.9)
Female	12 (35)	Other Saudi Hospitals	18 (37.5)
		Outside Saudi Arabia	7 (14.6)
**Regional Distribution**			
Riyadh	20 (41.7%)	**Level of spinal catheter tip**	
Asir	7 (14.6%)	Thoracic	23 (47.9)
Jazan	5 (10.4%)	Lumbar	5 (10.5)
Medina	4 (8.3%)	Unknown	20 (41.7)
Northern Borders Region	4 (8.3%)		
Eastern Region	3 (6.3%)		
Makkah	2 (4.2%)	**Boluses used for trial**	
Najran	1 (2.1%)	One	20 (41.7)
Al-Qassim	1 (2.1%)	Two	5 (10.4)
Al-Bahah	1 (2.1%)	Unknown	23 (47.9)
**Residence of patient**		**Pump Capacity**	
Within Riyadh City	17 (35.4)	20 mL	17 (35.4)
Outside Riyadh City	31 (64.6)	40 mL	31 (64.6)
**Diagnosis**		**ITB Related Complication**	
Spinal Cord Injury	32 (66.7)	Yes	17 (35.4)
Stroke	2 (4.2)	No	31 (64.6)
Traumatic Brain Injury	2 (4.2)		
Cerebral Palsy	8 (16.7)	**Primary Rehabilitation Physician**	
Demyelinating Disease	3 (6.3)	Spinal Cord Injury Rehabilitation	29 (60.4)
Anoxic Bain Injury	1 (2.1)	Neurorehabilitation	2 (4.2)
		Pediatric Rehabilitation	2 (4.2)
**Pattern of spasticity/dystonia**		Brain Injury Rehabilitation	4 (8.33)
Spastic Quadriparesis	24 (50)	None	11 (22.9)
Spastic Paraparesis	23 (47.9)		
Generalized Dystonia	1 (2.1)		

**Table 2:** Table showing scale data related to the patients and intrathecal baclofen pump.

Variables	Median (IQR)	Variables	Mean (SD)
Age (years)	30 (14)	Time since disease onset (months)	174.64 (60.99)
Number of years at King Fahad Medical City	8 (8)	Duration of ITB (months) since first ITB placement	119.45 (14.81)
Time since ITB trial to ITB placement (months)	1 (6)	Time since last baclofen pump placement (months)	38.64 (14.96)
Last appointment with primary physician (months)	23 (29)	Time since last ITB refill (months)	4.09 (1.45)
Baclofen dose (µg/day)	300.2 (305.45)	Estimated time for future refill (months)	3.9 (1.45)

**Table 3:** Complications and outcomes of intrathecal baclofen pump therapy.

Complications	Outcomes					Associated Surgery		
	Baclofen Withdrawal	Baclofen Overdose	Loss of Efficacy	Local Infection	Total n (%)	Pump Related Surgery	Catheter Related Surgery	Total n (%)
Pump infection	1	-	-	1	2 (11.1)	2	-	2 (20)
Programming error	3	1	-		4 (22.2)	-	-	-
Procedural complication (CAP * contrast study)		1	-	-	1 (11.1)	-	-	-
Catheter occlusion	3	-	1	-	4 (22.2)	-	4	4 (40)
Catheter breakage	-	-	2	-	2 (11.1)	-	2	2 (20)
Undetermined loss of efficacy (underwent surgical exploration)	-	-	2	-	2 (11.1)	2	-	2 (20)
Undetermined loss of efficacy (no surgical exploration) **	-	-	3	-	3 (16.6)	-	-	-
Total n (%)	7 (38.8)	2 (11.1)	8 (44.4)	1 (5.5)	18 (100)	4 (40)	6 (60)	10 (100)
**(%) of Total patients n = 48**	**14.5%**	**4.2%**	**16.7%**	**2.1%**	**37.5%**	**8.3%**	**12.5%**	**20.8%**

* Catheter access port (CAP); ** Patients underwent imaging evaluations, which were non-conclusive.

**Table 4:** Correlation of complications of intrathecal baclofen pump with different variables of interest.

Variable	Pump Infection n (%)	*p*-Value	Programming Error n (%)	*p*-Value	Procedural Complication (CAP * Contrast Study) n (%)	*p*-Value	Catheter Occlusion n (%)	*p*-Value	Catheter Breakage n (%)	*p*-Value	Undetermined Loss of Efficacy n (%)	*p*-Value
**Gender**												
Male	2 (5.6)	0.404	3 (8.3)	1	1 (2.8)	0.560	2 (5.6)	0.228	2 (5.6)	0.404	6 (17.1)	0.937
Female	-		1 (8.3)		-		2 (16.7)		-		2 (18.2)	
**Rehabilitation diagnoses**												
Spinal Cord Injury	2 (6.3)	0.959	3 (6.3)	0.596	(3.1)	0.992	2 (6.3)	0.576	2 (6.3)	0.959	4 (12.9)	0.209
Stroke	-		-		-		-		-		-	
Cerebral Palsy	-		-		-		2 (25)		-		2 (18.6)	
Demyelinating Disease	-		1 (2.1)		-		-		-		2 (66.7)	
Anoxic Brain Injury	-		-		-		-		-		-	
Traumatic Brain Injury	-		-		-		-		-		-	
**Spasticity pattern**												
Spastic Quadriparesis	-	0.322	3 (12.5)	0.573	-	0.574	3 (12.5)	0.001	1 (4.2)	0.978	3 (12.5)	0.361
Spastic Paraparesis	2 (8.7)		1 (4.3)		1 (4.3)		-		1 (4.3)		5 (22.7)	
Generalized Dystonia	-		-		-		1 (100)		-		-	
**Location of ITB pump placement**												
KFMC	1 (4.3)	0.952	1 (4.3)	0.338	1 (4.3)	0.292	2 (8.7)	0.931	1 (4.3)	0.952	5 (21.7)	0.437
Other Institutes	1 (4)		3 (12)		-		2 (8)		1 (4)		3 (13)	
**Living area of patient**												
Riyadh City	2 (11.8)	0.051	-	0.122	-	0.454	3 (17.6)	0.084	-	0.285	3 (20)	0.745
Outside Riyadh City	-		4 (12.9)		1 (3.2)		1 (3.2)		2 (6.5)		5 (16.1)	

* Catheter access port (CAP).

**Table 5:** Logistic regression analysis for possible risk factors for complications of intrathecal baclofen treatment.

Variables	Programming Error		Procedural Complication (CAP * Contrast Study)		Catheter Occlusion		Catheter Break		Undetermined Loss of Efficacy	
Omnibus Tests of Model Coefficients	χ^2^ = 10.210, *p* = 0.069		χ^2^ = 8.835, *p* = 0.116		χ^2^ = 8.835, *p* = 0.116		χ^2^ = 5.291, *p* = 0.381		χ^2^ = 5.488, *p* = 0.359	
Nagelkerke R^2^	52.3%		100%		100%		41.2%		27.7%	
Percentage Correct	90.3%		100%		100%		90.3%		80.6%	
**Variables in the Equation**	**Exp(B)**	***p*** **-value**	**Exp(B)**	***p*** **-value**	**Exp(B)**	***p*** **-value**	**Exp(B)**	***p*** **-value**	**Exp(B)**	***p*** **-value**
Age (years)	1.011	0.919	0.799	0.999	0.062	0.993	0.052	0.945	0.941	0.239
Distance from Riyadh (km)	1.001	0.690	1.056	0.997	0.876	0.993	0.875	0.355	1	0.897
Time since onset of primary disease (days)	1.065	0.371	1.173	0.998	0.966	0.995	3.277	0.392	0.986	0.162
Duration of ITB pump placement (months)	0.921	0.230	1.626	0.997	0.429	0.994	0.362	0.395	1.013	0.536
Baclofen dose per day (µg)	0.993	0.257	1.284	0.996	0.761	0.995	0.768	0.807	0.995	0.240

* Catheter access port (CAP).

## Data Availability

The data presented in this study are available upon request from the corresponding author.
